# Integrated GWAS, linkage, and transcriptome analysis to identify genetic loci and candidate genes for photoperiod sensitivity in maize

**DOI:** 10.3389/fpls.2024.1441288

**Published:** 2024-09-16

**Authors:** Yulin Jiang, Shuang Guo, Dong Wang, Liang Tu, Pengfei Liu, Xiangyang Guo, Angui Wang, Yunfang Zhu, Xuefeng Lu, Zehui Chen, Xun Wu

**Affiliations:** ^1^ Institute of Upland Food Crops, Guizhou Academy of Agricultural Sciences, Guiyang, Guizhou, China; ^2^ Ministry of Agriculture and Rural Affairs Key Laboratory of Crop Genetic Resources and Germplasm Innovation in Karst Region, Guiyang, China; ^3^ College of Agriculture, Guizhou University, Guiyang, China

**Keywords:** photoperiod sensitivity, genetic loci, GWAS, QTL, joint analysis, candidate gene

## Abstract

**Introduction:**

Maize photosensitivity and the control of flowering not only are important for reproduction, but also play pivotal roles in the processes of domestication and environmental adaptation, especially involving the utilization strategy of tropical maize in high-latitude regions.

**Methods:**

In this study, we used a linkage mapping population and an inbred association panel with the photoperiod sensitivity index (PSI) phenotyped under different environments and performed transcriptome analysis of T32 and QR273 between long-day and short-day conditions.

**Results:**

The results showed that PSIs of days to tasseling (DTT), days to pollen shedding (DTP), and days to silking (DTS) indicated efficacious interactions with photoperiod sensitivity for maize latitude adaptation. A total of 48 quantitative trait loci (QTLs) and 252 quantitative trait nucleotides (QTNs) were detected using the linkage population and the inbred association panel. Thirteen candidate genes were identified by combining the genome-wide association study (GWAS) approach, linkage analysis, and transcriptome analysis, wherein five critical candidate genes, *MYB163*, *bif1*, *burp8*, *CADR3*, and *Zm00001d050238*, were significantly associated with photoperiod sensitivity.

**Discussion:**

These results would provide much more abundant theoretical proofs to reveal the genetic basis of photoperiod sensitivity, which would be helpful to understand the genetic changes during domestication and improvement and contribute to reducing the barriers to use of tropical germplasm.

## 1 Introduction

Maize (*Zea mays* L.) originated in southern Mexico and has spread over a wide latitudinal range. In the process of expansion from tropical to temperate regions, maize germplasm has necessitated a reduction of its photoperiod sensitivity in adaptation to local environments (Edmeades et al., 2017). In southwest China, the resistance to diseases and insect pests was the most significant factor affecting maize production (https://www.stats.gov.cn/). The tropical maize germplasm was widely used during breeding practice, and the heterotic pattern of “temperate × tropical” played an important role in this region. However, tropical maize germplasms always showed stronger photoperiod sensitivity when planted under long-day (LD) environments, i.e., foundation inbred of T32 derived from tropical maize germplasms. Therefore, the photoperiod sensitivity characterization and relevant candidate genes are beneficial to major maize tropical germplasm improvement and seed production.

Most maize varieties are short-day (SD) plants, especially tropical germplasm, and the difference in photoperiod sensitivity largely reflects the responses of crop germplasm ecotypes with LD conditions (Birch et al., 1998; Khotasena et al., 2022). The photoperiod sensitivity index (PSI) could be a representative trait, which showed the stage of the plant to be affected by light length change from the vegetative to the reproductive stage. The specific calculation method is PSI = ± (average value of a trait under long day/average value under short day)/average value under short day (Zhang et al., 2011). Appropriate trait measurements are necessary to efficaciously appraise photoperiod sensitivity. Days to tasseling (DTT), days to pollen shedding (DTP), and days to silking (DTS) were successfully introduced for PSI consideration (Zhang et al., 2011; Khotasena et al., 2022; Wu et al., 2023). The leaf number (LN), plant height (PH), and ear height (EH) before the reproductive phase change are determined largely by the rate and pattern of leaf initiation during vegetative development and the duration of the vegetative phase controlled by the timing of floral transition (Langdale, 2005; Colasanti and Muszynski, 2009).

Several studies have detected quantitative trait loci (QTLs) for photoperiod sensitivity by using linkage analysis and association mapping. Li et al. (2016a) conducted a comprehensive genetic dissection to assess the genetic architecture of LN and its genetic relationship to flowering time, using a large set of 866 maize-teosinte BC_2_S_3_ recombinant inbred lines, and validated the pleiotropy of the genes *ZCN8*, *dlf1*, and *CCT* on LN and flowering. Li et al. (2016b) identified a total of 90 flowering time-related regions, found nearly 1,000 flowering time-associated single-nucleotide polymorphisms (SNPs), and mainly distributed approximately 220 candidate genes, by using the parallel linkage analysis of two NAM panels with more than 8,000 lines. Du et al. (2023) found a unique QTL *qPss3* by using two segregating populations that behaved as a dominant locus and caused earlier flowering by 2–4 days via inhibiting *ZmCCT10*-induced photoperiod sensitivity under LD conditions. Fei et al. (2022) identified 39 QTLs and explored four candidate genes (*Zm00001d006212*, *Zm00001d017241*, *Zm00001d047761*, and *Zm00001d047632*) enriched in the plant circadian rhythm pathway in the consensus major QTL region via combined analysis of transcriptome data. Wu et al. (2023) identified 25 important candidate genes for maize flowering time by integrating genome-wide association study (GWAS), linkage analysis, and transcriptome analysis. In conclusion, combining the method with linkage analysis and GWAS identified hundreds of candidate genes that may smash in the negative limitations of photosensitivity, thus contributing to the large-scale environmental adaptation of maize to altitude and latitude, such as *Vgt1*, *RAP2.7*, *Ghd7*, *D8*, *PHYC1/2/3*, *CCA1*, *Floridalma/Leafy 1/2*, *bif2*, CCTs, ZCNs, ZMMs, ELFs, and PRRs, to name a few (Xu et al., 2012; Wang et al., 2019; Zhao et al., 2023). It is worth noting that previous reports for photoperiod sensitivity aimed at flowering time, but few about PSI based on the values in different photoperiodic environments. These indirect traits, thermal time or PSI, have also true interactions with photoperiod sensitivity under LD or both LD and SD photoperiod environments (Xu et al., 2012).

In this study, we used a linkage mapping population with 150 F_2:3_ offspring obtained from the cross derived from the inbred lines T32 and QR273, and one natural association panel with 226 inbred lines to be materials, and proceeded PSI phenotyping under different photoperiod environments. Based on this, GWAS and linkage analysis were performed. After that, transcriptome data of T32 and QR273 were also determined between LD and SD conditions. Finally, QTL/quantitative trait nucleotides (QTNs) and relevant candidate genes for PSI of DTT, DTP, and DTS were identified. These results would help to provide some new proof for exploring the genomic basis of PSI in tropical maize germplasm.

## 2 Materials and methods

### 2.1 Plant materials and field experiments

An association mapping panel with 226 inbred lines and a linkage population with 150 F_2_ and F_2:3_ offspring derived from the cross between maize foundation inbred lines of QR273 and T32 were chosen in this study. Both foundation parents of QR273 and T32 were derived from the Suwan population. T32 was delayed in flowering and even failed to flower when planted under LD conditions, which showed a strong photoperiod sensitivity characteristic; on the contrary, QR273 bloomed normally (Wu et al., 2023). These materials were planted in Zhangye (ZY 38.93°N, 100.45°E, Gansu Province) for the strongest LD condition with an approximately 15-h/9-h photoperiod, Guiyang (GY 26.5°N, 106.7°E, Guizhou Province) for the mid-LD condition with a 13-h/11-h photoperiod, and Sanya (SY 18.36°N, 109.16°E, Hainan Province) for the SD condition with an approximately 12-h/12-h photoperiod. Meanwhile, the linkage population with F_2_ lines and the foundation parents QR273 and T32 were planted in SY for genotyping, and then F_2:3_ offspring and parent lines were planted in ZY and SY for phenotyping, respectively. A randomized complete block design was selected for field experiments, in which each line was planted in a single row, with a 3-m row length, with 12 individual plants in one row, and with an 80-cm space between rows. Field management including fertilization, irrigation, pest, and weed control was carried out based on local practices.

### 2.2 Photoperiod sensitivity index evaluation

LN, PH, EH, and three flowering time-related traits (DTT, DTS, and DTP) were chosen for PSI analysis. LN, PH, and EH were measured after the end of pollination. DTT, DTS, and DTP represent the number of days from sowing to the time when 50% of plants of each line exhibited the corresponding traits. PSI was calculated according to the method described by Fei et al. (2022): PSI = [(average value of the trait in long day − average value of the trait in short day)/average value of the trait in short day × 100%]. The PSI could be graded into four levels, in which <0% PSIs are classified as tolerant to the photoperiod, 0%–30% PSIs are identified as insensitive to the photoperiod, 31%–70% PSIs are considered as weakly sensitive to the photoperiod, and more than 71% PSIs are categorized as strongly photoperiod sensitive (Khotasena et al., 2022).

### 2.3 DNA extraction and genotyping

Leaf samples for DNA extraction were pooled from five plants in each line and collected from the five-leaf stage of maize seedlings. DNA extraction was performed according to the method following Saghai-Maroof et al. (1984). The association panel lines were genotyped using MaizeSNP50 BeadChip containing 56,110 SNPs, and this work was already done as described by Wu et al. (2019). Regarding linkage population lines, DNA quality testing and genotyping by sequencing (GBS) assessments were completed by the Beijing Compass Biotechnology Company, and the detailed methods followed the description by Wu et al. (2023).

### 2.4 Genome-wide association study and QTL mapping

The inbred association panel was utilized to perform a GWAS as described by Wang et al. (2023). Briefly, a total of 43,252 SNPs were used to perform a phenotype–genotype GWAS by using the TASSEL v5.2.80 software with a mixed linear model (MLM) and log10(*p*) threshold ≥4.0. The population structure and pairwise kinship were treated as covariates. Regarding the F_2:3_ population, after filtering SNPs with minor allele frequency (MAF < 0.05), we obtained a total of 68,994 high-quality SNPs that were used for the genetic map building. QTL mapping analyses were performed by using the QTL IciMapping software Version 4.1 (Wu et al., 2023). The positive correlations were detected by *r* > 0.8, *p* < 0.05.

### 2.5 Transcriptome analysis

The parent lines T32 and QR273 were grown at experiment fields of ZY and SY as described above. Leaf tissues were separated and pooled from three plants per line at the maize nine-leaf stage (V9) with three experimental repeats. Fresh tissues were immediately frozen in liquid nitrogen and stored at −80°C before RNA isolation. Total RNA was extracted using TRIzol (Invitrogen, Carlsbad, USA) according to the method by Gehrig et al. (2000). The cDNA synthesis and RNA sequencing were finished using the Illumina HiSeq 2000 platform at Biomarker Technologies Company (Beijing, China). The transcriptome data were analyzed as described by Wu et al. (2023).

### 2.6 Candidate gene prediction and verification

Functional annotations of these candidate genes were performed by using online search tools (https://www.maizegdb.org/). Quantitative reverse transcription polymerase chain reaction (qRT-PCR) analysis was performed as described by Jiang et al. (2021). The qRT-PCR primers were designed by the online PrimerQuest Tool (https://www.idtdna.com/Primerquest/Home/Index). ACTIN and GAPDH genes were used to normalize the expression of genes. The fold difference (2^−ΔΔCt^) and relative quantities were calculated using the CFX Manager Software version 3.1 (Bio-Rad, Hercules, USA).

### 2.7 Statistical analysis

Statistical analyses of experimental data were performed using the Student’s *t*-test (*p < 0.05, **p < 0.01, ***p < 0.001) by the software of IBM SPSS Statistics v22 (International Business Machines Corporation, New York, USA).

## 3 Results

### 3.1 PSI variation under different environments

**Figure 1:**
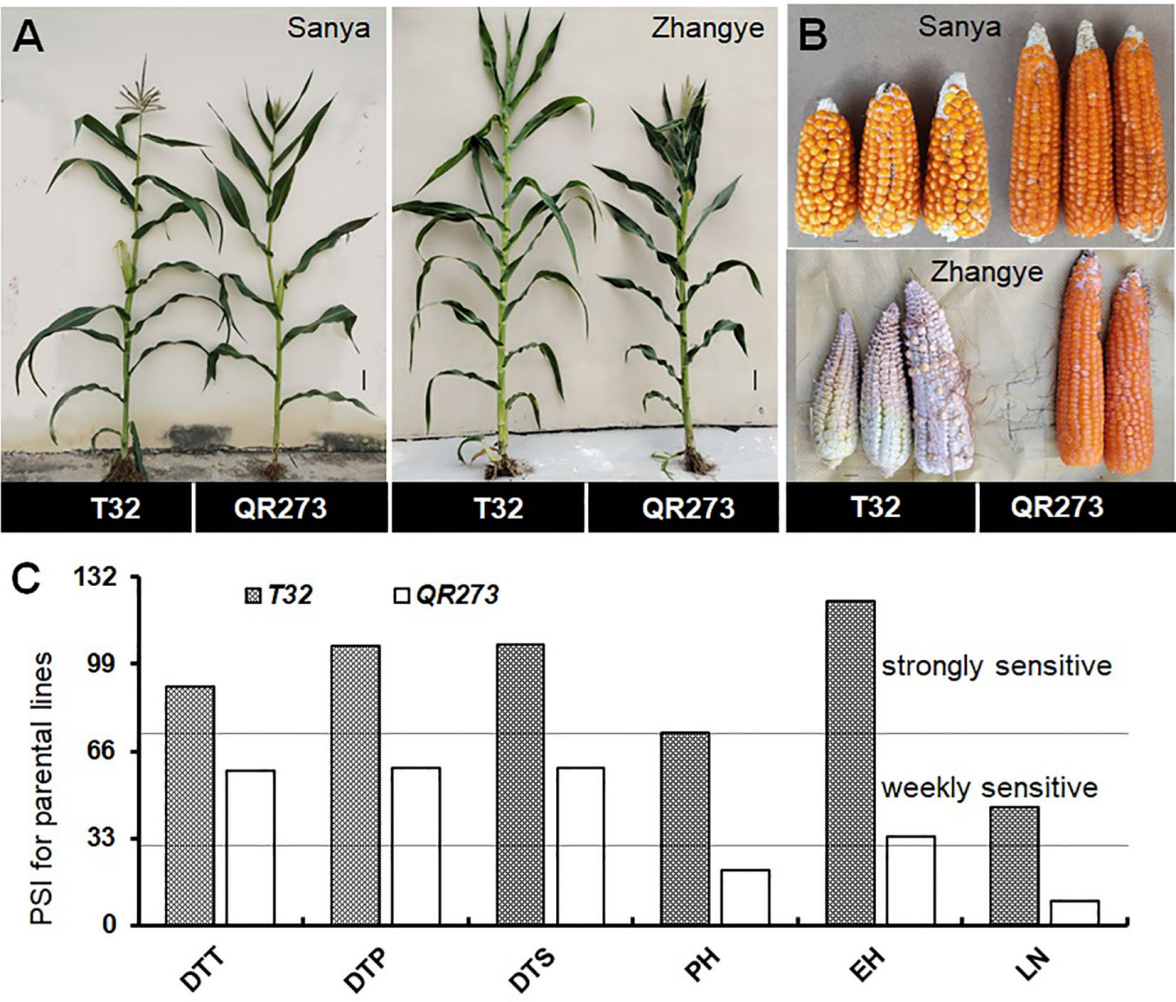
Linkage analysis for the photoperiod sensitivity index (PSI) of days to tasseling (DTT), days to pollen shedding (DTP), and days to silking (DTS), the leaf number (LN), plant height (PH), and ear height (EH) in the F_2:3_ population. The plant **(A)** and ear **(B)** phenotypes of the parental lines, T32 and QR273, in the different latitude areas (Sanya and Zhangye). **(C)** The PSI distribution of flowering time traits, PH, EH, and LN in the F_2:3_ population at the Sanya and Zhangye.

**Figure 2:**
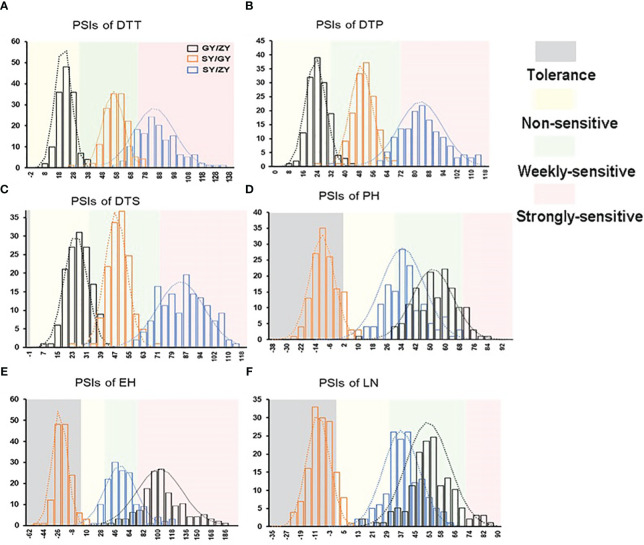
The histogram for PSIs of flowering time traits, **(A)** DTT, **(B)** DTP, **(C)** DTS, **(D)** PH, **(E)** EH, and **(F)** LN in the F2:3 population between pair-three latitude areas, including Guiyang vs. Zhangye (GY/ZY), Sanya vs. Guiyang (SY/GY), and Sanya vs. Zhangye (SY/ZY).

The tropical maize lines generally exhibit growth defects, such as delayed tasseling and silking, inconsistency between male and female, and yield decline when they are transplanted to high-latitude areas because of the high sensitivity characteristic to LD conditions. In this study, the foundation parental line T32 derived from the tropical germplasm of the Suwan population delayed tasseling, pollen shedding, and silking by approximately 54, 65, and 67 days, respectively, when it was grown at ZY (LD condition) compared to SY (SD condition). In addition, T32 also showed higher PH and EH, and more leaves (**Figure 1A**), but extremely fewer kernels (**Figure 1B**) when it was planted under LD conditions. Correspondingly, PSIs of DTT, DTP, DTS, PH, and EH for T32 were more than 71%, which indicated that these traits were highly associated with photoperiod sensitivity (**Figure 1C**). Although the foundation parental line QR273 also showed delayed flowering time-related traits under LD conditions when compared to those under SD conditions, PSIs of these traits were statistically lower and showed that such traits had weak or no photoperiod sensitivity (**Figure 1C**). After that, we analyzed the PSI of T32 and QR273, their F_2_ and F_2:3_ offspring, and an association panel planted under three different latitude areas with ~15 h/9 h (ZY), 13.5 h/10.5 h (GY), and 12 h/12 h (SY) photoperiods. Results showed that PSIs of most traits for SY/ZY showed a higher sensitivity for T32 and relevant F_2:3_ family lines, but a lower sensitivity for SY/GY and GY/ZY (**Table 1**). Additionally, PSIs of DTT, DTP, and DTS were highly consistent between pair-three latitude areas, with PSIs of SY vs. ZY (SY/ZY) above 90% (**Figures 2A–C**). Differently, PSIs of PH, EH, and LN for GY/ZY showed a higher discrepancy than those for SY/GY and SY/ZY (**Figures 2D–F**).

**Table 1:** The PSI analysis for QTL mapping population.

Environments	PSI of traits	Parental lines		F_2:3_ population lines		
		T32	QR273	Range	Mean ± SD	Coefficient of variation (%)
GY/ZY	DTT	26.40	12.50	17.28–34.66	20.39 ± 5.71	28.00
	DTP	31.44	12.21	18.02–32.97	22.61 ± 5.76	25.46
	DTS	32.34	14.45	17.98–32.29	25.19 ± 6.50	25.80
	PH	39.08	27.60	31.17–57.82	52.78 ± 11.32	21.46
	EH	99.70	59.35	45.23–164.04	102.50 ± 28.74	28.04
	LN	57.79	32.77	32.05–81.12	50.79 ± 12.21	24.03
SY/ZY	DTT	90.68	58.82	57.02–127.88	83.87 ± 14.19	16.92
	DTP	105.65	59.50	53.79–112.06	80.40 ± 12.76	15.87
	DTS	106.20	59.68	59.09–106.50	83.28 ± 13.23	15.88
	PH	72.83	20.70	32.02–41.21	36.50 ± 12.01	32.91
	EH	122.76	33.64	48.93–72.32	52.89 ± 19.59	37.03
	LN	44.64	8.97	33.77–54.35	36.71 ± 10.06	27.41
SY/GY	DTT	50.85	41.18	33.88–69.23	52.64 ± 7.33	13.93
	DTP	56.45	42.15	30.30–59.48	47.06 ± 6.25	13.28
	DTS	55.81	39.52	34.85–56.10	46.37 ± 6.15	13.27
	PH	24.26	−5.41	−10.53–0.65	−10.48 ± 6.90	65.82
	EH	11.55	−16.14	−34.74–2.55	−23.75 ± 9.54	40.19
	LN	−8.33	−17.93	−14.78–1.30	−9.06 ± 6.40	70.61

**Figure 3:**
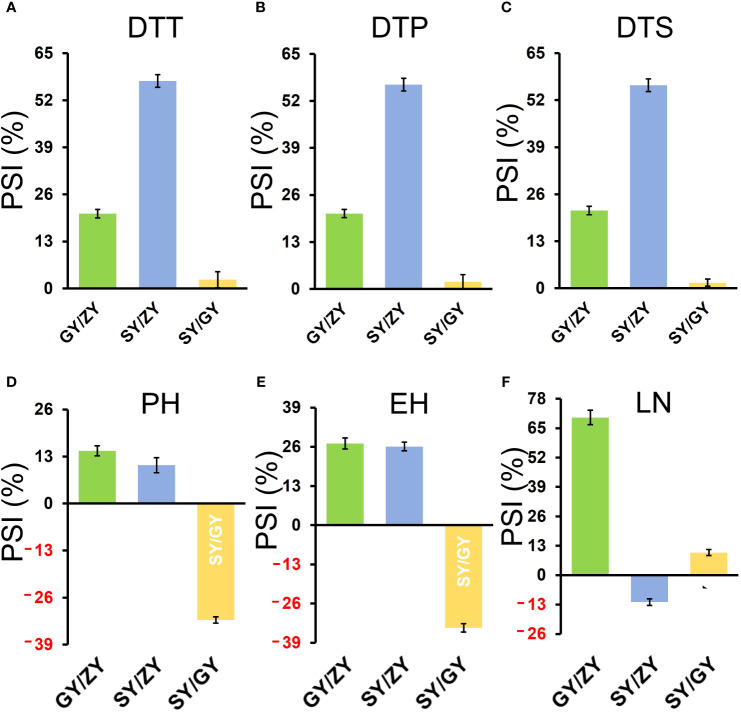
PSIs of DTT **(A)**, DTP **(B)**, DTS **(C)**, PH **(D)**, EH **(E)**, and LN **(F)** in the association mapping population between pair-three latitude areas, including GY/ZY, SY/GY, and SY/ZY. The Student’s *t*-test was introduced for statistical analysis.

For the association population, PSIs of DTT, DTP, and DTS were highly consistent between pair-three latitude areas (**Figure 3**). In addition, PSIs of DTT, DTP, and DTS for GY/ZY and SY/GY showed lower sensitivity than those for SY/ZY (**Figures 2**, **3**). However, PSIs of PH, EH, and LN appeared to vary inversely from those of flowering time traits. For the linkage population, PSIs of PH and LN for GY/ZY and SY/ZY showed weak sensitivity and tolerance for SY/GY; meanwhile, PSIs of EH for SY/GY, SY/ZY, and GY/ZY showed tolerance, weak sensitivity, and strong sensitivity, respectively (**Figures 2D–F**). The average PSIs of PH and EH for the association panel were similar between GY/ZY and SY/ZY, but PSIs of LN for SY/GY showed tolerance (**Figures 3D–F**). Moreover, we found that most tropic lines, such as 7031, QB2182, QB2208, S909, ZH6218, and CML171 (above 60%), have higher PSI values than temperate maize lines.

### 3.2 QTL analysis for PSI

PSIs of DTT, DTP, and DTP for F_2_ and F_2:3_ family lines showed rich variation under three different altitudes with values ranging from 20.39% (GY/ZY) to 83.87% (SY/ZY) (**Table 1**). The significantly positive correlations were detected between pair-PSIs of three flowering-related traits (**Supplementary Table S1**). The skewness and peak results were consistent with normal distribution (**Figure 2**), which could be used for further QTL analysis. A total of 68,994 SNP markers were used to construct the genetic linkage map, and QTL mapping is presented in **Supplementary Figure S1**. A total of 48 QTLs related to flowering time-related trait PSIs were identified and explained 3.67%–17.59% of phenotypic variation (PVE) (**Supplementary Table S2**). Among them, 17 QTLs showed PVEs of more than 10%, and 7 QTLs showed PVEs of less than 5%, indicating that PSIs for flowering time were controlled by both minor and multiple loci.

For PSIs of DTT, six, four, and three QTLs were found for SY/GY, GY/ZY, and SY/ZY, respectively. Regarding PSIs of DTP, a total of 18 QTLs were detected between three different altitude areas, with 6, 5, and 7 QTLs to be found for SY/GY, GY/ZY, and SY/ZY, respectively. In addition, for PSIs of DTS, 2, 5, and 10 QTLs were found for SY/GY, GY/ZY, and SY/ZY, respectively. Among these QTLs, three of them exhibited pleiotropic effects: (1) both *qPSI_DTS2_4–1* from GY/ZY and *qPSI_DTT3_4–1* from SY/ZY were located at 133.4 Mb on Chr4 (**Supplementary Table S2**, orange blocks); (2) *qPSI_DTP1_7–3*, *qPSI_DTP3_7–1*, and *qPSI_DTS3_7–3* were positioned at 184.1 Mb on Chr7 (**Supplementary Table S2**, blue blocks); and (3) *qPSI_DTP2_9–1* and *qPSI_DTS2_9–1* were located at 42.5 Mb on Chr9 (**Supplementary Table S2**, gray blocks). It should be noted that *qPSI_DTP1_9–1* and *qPSI_DTP3_9–1* for DTP were detected for both SY/GY and SY/ZY, which were localized at 190.5 Mb on Chr9, with 13.23% and 11.98% of high PVE, respectively (**Supplementary Table S2**, green blocks). Both *qPSI_DTP1_7–3* and *qPSI_DTP3_7–1* were found for both SY/GY and SY/ZY, which were also positioned in the same regions (**Supplementary Table S2**, blue blocks).

In these QTLs, a total of 316 genes were found, including 64 candidate genes with annotated functions (**Supplementary Table S3**). Several important genes related to photoperiod sensitivity or flowering time-related traits were found, e.g., *Zm00001d051294* at *qPSI_DTS3_4–1* encodes the NAC-transcription factor 27, *Zm00001d012255* at *qPSI_DTS3_2–2* encodes the MYB-transcription factor, *Zm00001d022353* at *qPSI_DTS3_7–2* encodes the BSD-transcription factor 7, *Zm00001d001894* at *qPSI_DTS3_2–2* encodes the Trihelix-transcription factor 35, *Zm00001d020256* at *qPSI_DTT2_7–1* is the dark response gene 15, *Zm00001d037749* at *qPSI_DTS1_6–1* encodes the bHLH-transcription factor 124, and *Zm00001d017660* at *qPSI_DTT1_5–3* encodes Protein photoperiod-independent early flowering 1 (**Supplementary Table S3**). The Gene Ontology (GO) enrichment analysis indicated that 14 GO terms of most candidate genes are associated with cell, intracellular, and membrane-bounded organelle (**Supplementary Figure S2A**). In addition, the Kyoto Encyclopedia of Genes and Genomes (KEGG) analysis detected seven significant terms involving metabolic pathway, biosynthesis of amino acids, lysine, and monobactam (**Supplementary Figure S2B**).

### 3.3 GWAS for PSI

**Figure 4:**
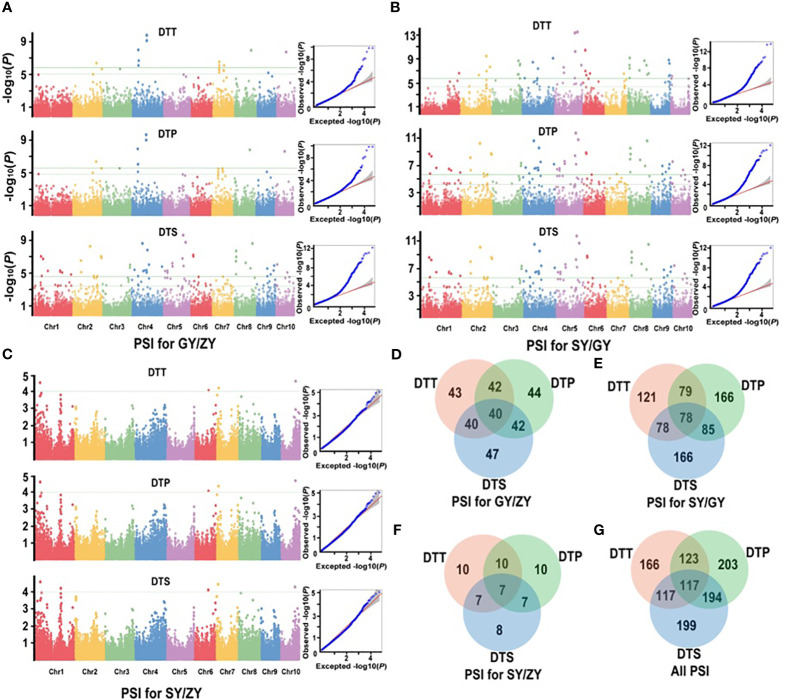
Manhattan and Q-Q plots from a mixed linear model for maximum quantum efficiency for PSIs of flowering time traits in the association mapping population. **(A)** PSIs for GY/ZY, **(B)** PSIs for SY/GY, and **(C)** PSIs for SY/ZY. **(D–F)** show the common significant QTNs of PSIs between pair-three latitude areas, respectively. **(G)** shows that the common significant QTNs were collectively related to PSIs in three comparisons.

For the association population, a significantly positive correlation was also observed between PSIs of DTT, DTP, and DTS under three specific environments (**Supplementary Table S4**). A total of 252 significant QTNs were identified (**Supplementary Table S5**), with 49, 212, and 12 QTNs in GY/ZY, SY/GY, and SY/ZY, respectively (**Figures 4A–C**). Additionally, 43, 44, and 47 QTNs were found to be related to DTT, DTP, and DTS of PSIs for GY/ZY, in which 40 QTNs were related to all three traits (**Figure 4D**). Regarding PSIs for SY/GY, a total of 121, 166, and 166 QTNs were found to be associated with DTT, DTP, and DTS, respectively, wherein 78 QTNs were composed of all three traits (**Figure 4E**). Notably, only 12 QTNs were found to be related to PSIs for SY/ZY, wherein, 10, 10, and 8 were associated with DTT, DTP, and DTS, respectively (**Figure 4F**). Meanwhile, seven QTNs were found to be simultaneously related to DTT, DTP, and DTS traits (**Figure 4F**). These QTNs explained 9.0%–33.1% of phenotypic variation, wherein QTN of SYN1149 located on Chr5 was significantly associated with DTT, DTP, and DTS for both SY/GY and GY/ZY, which can explain 33.1% of phenotypic variation for DTT of PSI at SY/GY (**Supplementary Table S5**). In addition, a total of 166, 203, and 199 QTNs associated with PSI of DTT, DTP, and DTS were found, respectively (**Figure 4G**). Meanwhile, 117 important common QTNs were found to be related to the three traits DTT, DTP, and DTS (**Figure 4G**).

Among the loci mentioned above, 5,084 candidate genes were found (**Supplementary Table S6**), wherein 17, 55, 192, and 785 genes were found to be linked to five, four, three, and two SNPs, respectively (**Supplementary Table S6**). Furthermore, these candidate genes were annotated to be tRNA, lincRNA, miRNA, and transcription factors, and involved in synthetase, transferases, and binding protein function. Among these genes, *Zm00001d008700* encodes a grass-specific tryptophan aminotransferase required for vegetative and reproductive development in maize (Phillips et al., 2011), *Zm00001d021826* belongs to GRAS transcription factors that have potential roles in growth and development (Kumari et al., 2023), and *Zm00001d051012* encodes a leucine-rich repeat receptor-like protein that regulates shoot meristem proliferation in maize and determines inflorescence meristem size and yield traits in barley (Taguchi-Shiobara et al., 2001; Wang et al., 2023). In addition, the GO enrichment analysis indicated that 148 significant (*p* < 0.001) GO terms were associated with important biological processes, including transcription factor, solute antiporter, hydron symporter, and glucose transmembrane transporter activities (**Supplementary Figure S3**; **Figure 4**).

### 3.4 Transcriptome analysis

**Figure 5:**
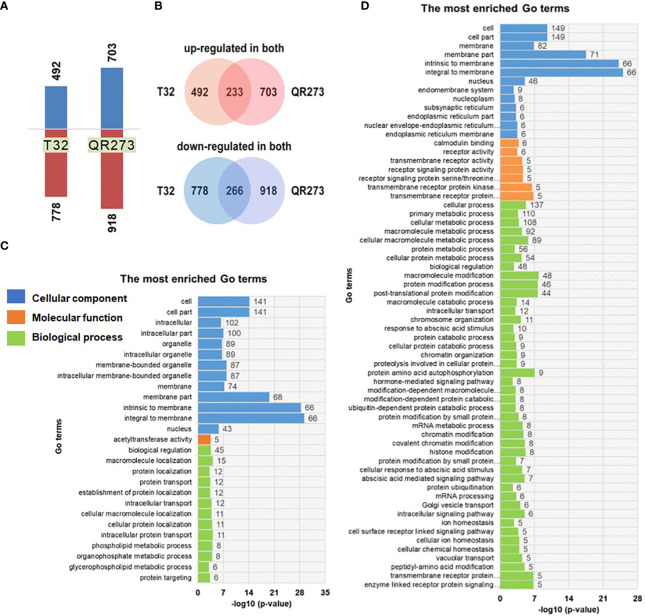
The differentially expressed genes (DEGs) identified between the two foundation inbred lines of T32 and QR273 in long-day (Zhangye) and short-day (Sanya) environments. **(A)** The number of upregulated and downregulated DEGs by long-day condition. **(B)** The common upregulated and downregulated DEGs in T32 and QR273. **(C, D)** The most enriched GO terms for the upregulated **(C)** and downregulated **(D)** genes.

Transcriptome analysis of T32 and QR273 showed a number of differentially expressed genes (DEGs) involved in photoperiod sensitivity (**Figure 5**). For T32, a total of 9,343 genes were co-expressed in both SD and LD conditions, and 1,901 genes were significantly differentially expressed, including 492 genes significantly upregulated and 778 genes significantly downregulated under LD conditions in comparison to those under SD conditions (**Figure 5A**). Meanwhile, a total of 2,963 genes were co-expressed in QR273 under both conditions, including 704 genes significantly upregulated and 916 genes significantly downregulated under LD conditions when compared to those under SD conditions (**Figure 5B**). Among these DEGs, 233 and 266 genes were simultaneously upregulated and downregulated for both T32 and QR273, respectively (**Figure 5B**).

Then, GO enrichment analyses were implemented to predict the biological processes and functions of these DEGs (**Figures 5C, D**). A total of 27 and 64 significant terms were detected in the upregulated and downregulated genes, respectively. Among the upregulated genes, 58, 45, and 74 genes were involved in oxidoreductase activity, biological regulation, and membrane components, respectively (**Figure 5C**). Regarding the downregulated genes, seven and eight genes individually participated in abscisic acid (ABA)-mediated and hormone-mediated signaling pathway, five genes were regulated by transmembrane receptor protein serine/threonine kinase activity, and nine genes were localized at the endomembrane system (**Figure 5D**). These results seem to imply a significant difference between the upregulated and downregulated DEGs. Furthermore, the downregulated genes are involved in four important biological processes, namely, protein ubiquitination, ubiquitin-dependent protein catabolic process, protein amino acid autophosphorylation, and ABA-mediated signaling pathway (**Supplementary Figure S5**).

### 3.5 Candidate genes analysis

**Figure 6:**
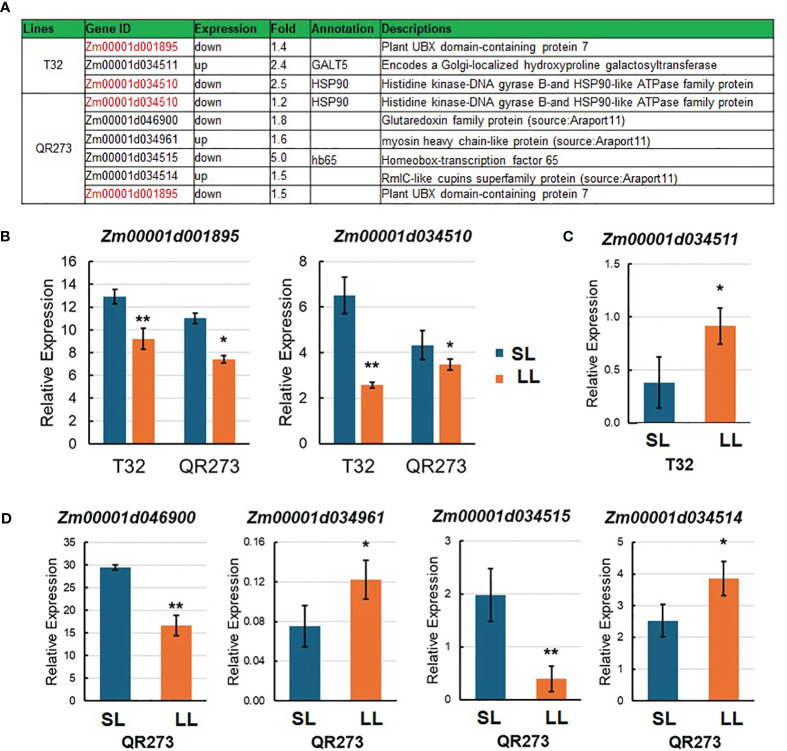
Information of candidate genes identified by genome-wide association study (GWAS) and transcriptome analysis. **(A)** Annotation information of seven selected candidate genes. Red text indicates DEGs in both T32 and QR273. **(B–D)** show the relative expression levels of candidate genes in T32 and/or QR273 by qRT-PCR. **(B)** The relative expression of *Zm00001d001895* and *Zm00001d034510* in both T32 and QR273, **(C)** the expression of *Zm00001d034511* in T32, and **(D)** the expression of *Zm00001d034961*, *Zm00001d034514*, *Zm00001d046900*, and *Zm00001d034515* in QR273. Asterisks (*) indicate statistically significant differences between long-day (LD) and short-day (SD) conditions (*p < 0.05, **p < 0.001).

In this study, seven candidate genes were identified when comparing the results of linkage mapping and transcriptome analysis (**Figure 6A**). Here, *Zm00001d001895* and *Zm00001d034510* were downregulated for both T32 and QR273 planted under an LD environment, and these results were proven by qRT-PCR (**Figure 6B**). *Zm00001d001895* encodes the plant Ubiquitin Regulatory X (UBX) domain-containing protein 7 (PUX7), which functions as a negative regulator of gibberellin (GA) signaling involved in seed germination, the transition to flowering, and cell elongation and division (Hauvermale et al., 2022). *Zm00001d034510* encodes the Histidine kinase-DNA gyrase B-and HSP90-like ATPase family protein (HSP90). Its homologous genes from Arabidopsis, AtHSP90.7, have important roles in the correct folding and/or the formation of the CLV1/CLV2 transmembrane-type receptor complex that is essential for shoot and floral meristem development (Kadota and Shirasu, 2012). In addition, *Zm00001d034511* of T32 was upregulated when planted under LD conditions (**Figure 6C**), and it encodes a Golgi- localized hydroxyproline galactosyltransferase (GALT5). The homologous gene from Arabidopsis, At*GALT2*, controls AGP (arabinogalactan-proteins) O-glycosylation, which was essential for normal growth and development (Basu et al., 2015). Among the four genes of QR273, *Zm00001d034961* and *Zm00001d034514* were significantly upregulated when planted under LD conditions; in contrast, *Zm00001d046900* and *Zm00001d034515* were downregulated (**Figure 6D**).

**Figure 7:**
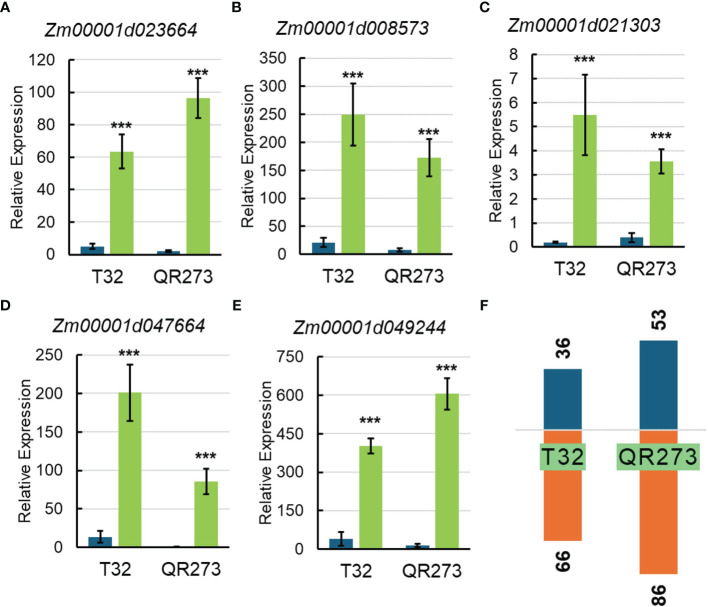
The relative expression of key candidate genes selected by GWAS and transcriptome analysis and the number of DEGs in T32 and QR273. **(A–E)** The relative expression levels of key candidate genes including *Zm00001d023664* **(A)**, *Zm00001d008573* **(B)**, *Zm00001d021303* **(C)**, *Zm00001d047664* **(D)**, and *Zm00001d049244* **(E)**. Asterisks (*) indicate remarkably significant differences between long-day (LD) and short-day (SD) conditions (***p < 0.0001). **(F)** The number of upregulated and downregulated DEGs in T32 and QR273.

Among the 5,084 genes identified by using GWAS, 203 genes were significantly and differentially expressed under LD conditions when compared to those under SD conditions. We found that 102 and 139 genes were specifically found for T32 and QR273 when planted under LD conditions. A total of 38 genes were commonly regulated for both T32 and QR273, and 15 and 23 genes were upregulated and downregulated by LD conditions in both T32 and QR273, respectively (**Table 2**). Notably, five genes were remarkably induced in both T32 and QR273 when plants were grown under an LD environment, namely, *Zm00001d023664*, *Zm00001d008573*, *Zm00001d021303*, *Zm00001d047664*, and *Zm00001d049244* (**Figures 7A–E**). *Zm00001d023664* was involved in the hormone that could relate to plant flowering time. *Zm00001d008573* (*CaDK2C*) encoded calmodulin protein 2 and was an ortholog with Arabidopsis *AtCML23* gene (*AT1G66400*), which regulates nitric oxide levels and transition to flowering (Tsai et al., 2007; Dou et al., 2024). In addition, 36 and 66 genes from T32 were respectively upregulated and downregulated when planted under LD conditions (**Figure 7F**). Meanwhile, 53 and 86 genes from QR273 were remarkably upregulated and downregulated when planted under LD conditions, respectively (**Figure 7F**).

**Table 2:** Annotation information of 38 selected candidate genes by GWAS and transcriptome data.

Genes	Expression	Fold	Expression	Fold	Annotation	Description
	T32		QR273			
Zm00001d035481	Down	1.62	Down	2.70	MYND	Programmed cell death protein 2 C-terminal domain-containing protein
Zm00001d006030	Down	1.29	Down	1.34	ENT	ENT domain containing protein
Zm00001d033860	Down	2.08	Down	2.58		Uncharacterized
Zm00001d034370	Down	2.43	Down	3.29		F-type ATPase gamma/ML domain protein
Zm00001d007214	Down	1.61	Down	1.15	TIM44–2	Mitochondrial import inner membrane translocase subunit TIM44–2
Zm00001d003904	Down	2.13	Down	1.52	TRAPP	Transport protein particle (TRAPP) component
Zm00001d008708	Down	2.08	Down	1.94	DUF	Putative DUF1421 domain family protein
Zm00001d008743	Down	1.93	Down	1.23		Ubiquitin carboxyl-terminal hydrolase
Zm00001d008748	Down	2.50	Down	2.21		Uncharacterized
Zm00001d011927	Down	3.34	Down	5.88		Uncharacterized
Zm00001d012307	Down	5.67	Down	1.96	nbcs22	Nucleobase: cation symporter 22
Zm00001d013336	Down	1.53	Down	1.52	BED	Zinc finger BED domain-containing protein DAYSLEEPER
Zm00001d013712	Down	2.28	Down	1.92	FLK	Flowering locus K homology domain
Zm00001d021595	Down	1.91	Down	1.27		Uncharacterized
Zm00001d027503	Down	2.07	Down	2.20	CBEF	Calcium-binding EF hand family protein
Zm00001d036055	Down	2.91	Down	2.13		Uncharacterized
Zm00001d037058	Down	1.45	Down	1.57	CAK1AT	Encodes a CDK-activating kinase that regulates root initial cell differentiation
Zm00001d037604	Down	2.30	Down	2.99	ETR3	Ethylene receptor homolog 3
Zm00001d037606	Down	2.03	Down	2.14	imp2/ARM42/IMPa3	Importin 2/Importin subunit alpha
Zm00001d039341	Down	3.34	Down	2.14	EIN2	Ethylene insensitive 2
Zm00001d044188	Down	2.34	Down	1.51		Glycosyl transferase family 1 protein
Zm00001d048224	Down	2.19	Down	2.62	VQ57	VQ motif-transcription factor 57
Zm00001d053701	Down	6.60	Down	6.53		Nuclear pore complex protein NUP 96
Zm00001d028948	Up	2.47	Up	4.63	cal3	Calmodulin 3
Zm00001d028941	Up	10.99	Up	4.35	vp3	VQ motif-transcription factor 3
Zm00001d034372	Up	3.39	Up	5.47	cdpk50/cpk11	Calcium-dependent protein kinase 50
Zm00001d023664	Up	12.62	Up	49.04		ABA-responsive protein
Zm00001d008573	Up	11.68	Up	20.94	CaDK2C	Calmodulin protein 2, touch-induced
Zm00001d044033	Up	2.95	Up	2.50	YLS7	Trichome birefringence-like 20/tbl17, yellow-leaf-specific gene 7
Zm00001d011256	Up	2.02	Up	1.50	ohp3	Opaque 2 heterodimerizing protein 3
Zm00001d013333	Up	3.32	Up	12.51		Uncharacterized
Zm00001d016591	Up	1.65	Up	1.93		Serine/threonine-protein kinase PBS1; probable serine/threonine-protein kinase PBL7
Zm00001d020100	Up	2.56	Up	3.83	prh6/PP2C-A4	Protein phosphatase homolog 6
Zm00001d021303	Up	28.15	Up	9.12		Uncharacterized
Zm00001d031807	Up	6.79	Up	5.68		Solute carrier family 25 member 44
Zm00001d046471	Up	7.26	Up	16.50	mbf2	Multi-protein bridging factor homolog 2
Zm00001d047664	Up	14.85	Up	97.67		Uncharacterized
Zm00001d049244	Up	9.90	Up	40.99	BAG	BAG domain-containing protein

**Figure 8:**
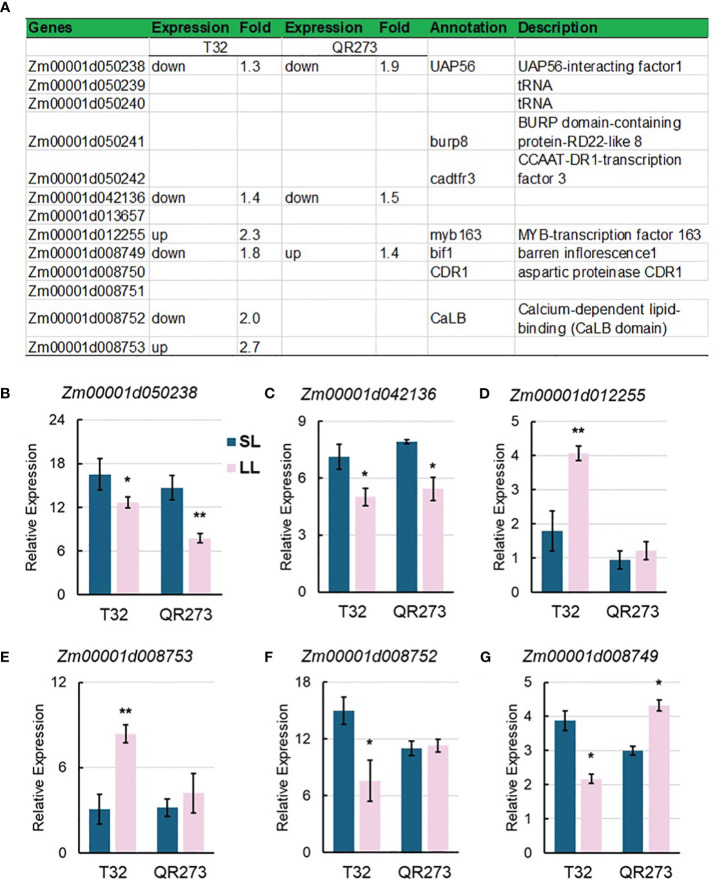
Information of candidate genes identified by QTL and GWAS analysis. **(A)** Annotation information of 13 selected candidate genes. **(B–D)** show the relative expression levels of main candidate genes by qRT-PCR including *Zm00001d050238* **(B)**, *Zm00001d042136* **(C)**, *Zm00001d012255* **(D)**, *Zm00001d008753* **(E)**, *Zm00001d012252* **(F)**, and *Zm00001d008749* **(G)**. Asterisks (*) indicate statistically significant differences between long-day (LL) and short-day (SL) conditions (*p < 0.05, **p < 0.001).

Four major QTLs, *qPSI_DTP1_4–1*, *qPSI_DTP2_3–2*, *qPSI_DTP3_5–1*, and *qPSI_DTP3_8–1*, were also identified by using GWAS, including 13 overlapped genes (**Figure 8A**), wherein *Zm00001d050238* and *Zm00001d042136* were downregulated in both T32 and QR273 planted under LD conditions (**Figures 8B, C**). *Zm00001d012255* and *Zm00001d008753* were upregulated in T32 but did not respond in QR273 when planted under LD conditions (**Figures 8D, E**); in contrast, *Zm00001d012252* was downregulated in T32 but did not respond in QR273 (**Figure 8F**). Interestingly, *Zm00001d008749* was downregulated in T32 but upregulated in QR273 when planted under LD conditions compared to under SD conditions (**Figure 8G**).

## 4 Discussion

### 4.1 PSI is a key indicator for dissecting the genetics of maize photoperiod sensitivity

Flowering time, including DTT, DTP, and DTS, is a proper trait for PSI measurements and can be used to evaluate the photoperiod sensitivity caused by latitude discrepancy. In the current study, PSIs of DTT, DTP, and DTS analysis were highly consistent between pair-three latitude areas. However, PSIs of PH, EH, and LN showed different variants when compared to flowering time traits (**Figures 2**, **3**). These results seemed to mean that PSIs of PH, EH, and LN did not directly reflect the influences of photoperiod. Moreover, PSIs of DTT, DTP, and DTS for GY/ZY and SY/GY were lower than those for SY/ZY (**Figures 2**, **3**), which meant that PSI values increase with incremental latitude gap or photoperiod length. Moreover, most tropical lines, such as 7031, QB2182, QB2208, S909, ZH6218, and CML171, have higher PSI values than temperate maize lines, meaning that tropical maize basically shows more serious photoperiod sensitivity than temperate maize. Li et al. (2020), by using 39,350 high-quality SNP markers in temperate and tropical maize groups, showed that with decreased latitude, the number of days to flowering was shortened for both temperate and tropical inbred lines, and temperate maize showed a greater difference in longer days. These results were similar to our findings.

Furthermore, QTL for the PSIs in the current results is in line with only six co-located loci in our previous study that are associated with QTL for flowering time traits (Guo et al., 2023), which meant that QTL for the PSIs is independent of the QTL for direct flowering time traits. These results were also similar to the present finding by Sabeti et al. (2002) and Li et al. (2020). Consistently, Fei et al. (2022) and Wu et al. (2023) successfully used PSI of flowering time traits to obtain significant loci and predict candidate genes. These studies mean that PSI, as an indirect trait, was also suitable for mining candidate genes, and these studies will help to understand the genetic changes during domestication and improvement and contribute to reducing the barriers to the use of tropical germplasm.

### 4.2 PSI related genetic loci

As described above, the six QTLs in line with our previous study were *qPSI_DTT1_9–1*, *qPSI_DTP2_7–1*, *qPSI_DTS2_4–1*, *qPSI_DTP3_4–1*, *qPSI_DTT3_4–1*, and *qPSI_DTS3_7–2* (Guo et al., 2023). *qPSI_DTP3_4–1* and *qPSI_DTT3_4–1* share the same region on Chr4 and are associated with two candidate genes, *Zm00001d050948* and *Zm00001d050952* (**Supplementary Table S2**). The two genes individually encode AP2-EREBP-transcription factor 132 (*ereb132*) and ZIM-transcription factor 49 (*zim49*), and both of them are involved in plant development, hormones, and blooming (Dietz et al., 2010; Liu et al., 2017). Furthermore, *qPSI_DTT1_5–2* overlapped with qPHPS1–2 associated with PSI of PH in Fei et al. (2022). In *qPSI_DTT1_5–2*, a candidate gene, *Zm00001d016080*, was found to encode an Extra-large G-protein-like protein (XLG); is involved in maize and rice growth, development, and stress responses; and regulates agronomic traits such as PH, tiller, panicle number, heading time, and grain size/shape (Cantos et al., 2023). These results suggested that the seven QTLs may be hotspot regions related to maize photoperiod sensitivity. Among the total 64 annotated genes detected in this paper, 3 known flowering time-related genes, *ZCN4* (*Zm00001d003804*) belonging to FLOWERING LOCUS T (FT) members, *BURP8* (*Zm00001d050241*), and *AVT1I* (*Zm00001d037229*), can regulate maize flowering (Azodi et al., 2019; Su et al., 2024). *Zm00001d017660* is annotated as protein photoperiod-independent early flowering 1.

### 4.3 QTNs related to PSI

In maize, several studies were conducted to identify QTNs and candidate genes involved in photoperiod sensitivity through GWAS analysis. Yang et al. (2013) performed a GWAS using 368 inbred lines of maize and identified 48 polymorphic sites associated with flowering time, in which 42 of the 48 sites were located within the promoter region of the *ZmCCT* gene that was an important gene that attenuated photoperiod sensitivity. Li et al. (2020) revealed 106 selective-sweep regions containing 423 candidate genes using 39,350 high-quality SNP markers in temperate and tropical maize groups consisting of 410 inbred lines phenotyping in three representative experiments in different latitudes.

In this study, a total of 252 SNPs and 5,084 genes within a 1-Mb region (Li et al., 2016a) for PSI of the flowering time traits were found in maize planting in different latitude areas. Compared to our previous studies, one (PZE-102181889) and four (PZE-108077370, SYN4993, SYN34850, and PZE-109065940) common SNPs were found in Wu et al. (2023) and Li et al. (2016b), respectively. Among these candidate genes associated with the above SNPs, Zm00001d007191 belongs to the MYB-transcription family this family has proven functions for plant flowering (Liu et al., 2014; Zhu et al., 2020; Hong et al., 2021; Wu et al., 2023). *Zm00001d007188*, belonging to Ethylene-insensitive 3 (EIN3)/EIN3-Like (EIL) transcription factors, is a core component of the ethylene signaling pathway that plays important roles in plant development and flowering (Mao et al., 2022). *Zm00001d023592*, *Zm00001d023593*, and *Zm00001d023596* encode the amino acid/auxin permease 64, 65, and 66 (*aaap64/65/66*), respectively. Their homology *AAP2/3/5* from Arabidopsis participated in the high-affinity transport system for acidic and neutral amino acids in the tapetum cells of Arabidopsis flowers (Lee and Tegeder, 2004). Interestingly, a small auxin-up RNA gene (*SAUR*) cluster was in the region of SYN34850, including *SAUR65*, *66*, *67*, and *68*, and many previous studies have shown that *SAUR* genes play important roles in heading date, flower morphogenesis, and pollination by inducing auxin (Kant et al., 2009; Ke et al., 2018; Sun et al., 2023). For example, *SAUR63* promotes hypocotyl and stamen filament elongation in Arabidopsis (Chae et al., 2012); *SAUR62/75* are required for the translation of transcripts essential for pollen tube growth (He et al., 2018); *SAUR7* possibly regulates the development of flower organs in apples (Wang et al., 2020); *SAUR56* regulates heading date in rice (Zhao et al., 2023).

### 4.4 Common genetic loci related to PSI via combined analysis

The joint analysis of combining multiple methods, such as the GWAS approach, linkage analysis, and transcriptome analysis, to quickly find key candidate genes has proven to be effective. Hung et al. (2012) found 25 candidate genes, including important and known flowering genes *IF-4A*, *Id1*, and *Vgt1*, mapped within both joint linkage QTL and GWAS of photoperiod sensitivity for thermal time to silking in maize NAM and IBM populations. Similarly, Li et al. (2021) identified the *WRKY14* gene by combining GWAS and linkage analysis, which is a critical regulator of maize flowering time and LN. In this study, 13 overlapped genes were found by integrating the results of GWASs, QTL mapping, and transcriptome analysis, and five putative key candidate genes (*MYB163*, *BIF1*, *BURP8*, *CADR3*, and *Zm00001d050238*) were selected.

In detail, *Zm00001d012255* encodes MYB-transcription factor 163, and MYB proteins are key factors in regulatory networks controlling the development, metabolism, and responses to biotic and abiotic stresses (Dubos et al., 2010). For example, *PtrMYB192*, a *Populus* R2R3 MYB transcription factor, regulates flowering time in Arabidopsis by activating FLOWERING LOCUS C (Liu et al., 2013). The overexpression of an apple R2R3 MYB transcription factor, *MdMYB3*, has resulted in transcriptional activation of several flavonoid pathway genes and produced longer peduncles and pistil styles of flowers than those of wild-type plants, meaning that it is involved in the regulation of flower development (Vimolmangkang et al., 2013). *Zm00001d008749* was remarked to be associated with the mutant *bif1* that causes barren inflorescence. Previous research found that two BIF genes, BIF1 and BIF4, encode AUXIN/INDOLE-3-ACETIC ACID (Aux/IAA) proteins and regulate the early steps required for inflorescence formation by the auxin hormone signaling pathway (Galli et al., 2015). Notably, *Zm00001d050238* was reported to be involved in the *cis*-regulation underlying maize highland adaptation that is also highly related to traits like maize flowering time (Hu et al., 2022).

In addition, the two genes *Zm00001d050241* and *Zm00001d050242* may be indirectly related to maize flowering. *Zm00001d050241* encodes BURP domain-containing protein-RD22-like 8 (*BURP8*), in which BURP genes are differentially responsive to ABA and ABA-related stress conditions (Matus et al., 2014). *ZmBURP8* participates in the ABA-responsive pathway by *ZmPRR37* regulation, in which ZmPRR37-CR knockout mutants exhibited early flowering compared to WT under LD conditions (Su et al., 2024). *Zm00001d050242* encodes CCAAT-DR1-transcription factor 3 (CADR3) and belongs to the NUCLEAR FACTOR-Y (NF-Y) family; NF-Ys have been shown to play an important role in growth, development, and the response to environmental stress (Zhang et al., 2016). Those potential candidate genes via joint analysis would greatly advance our understanding of maize photoperiod sensitivity.

### 4.5 Conclusion

In conclusion, PSIs of DTT, DTP, and DTS showed efficacious interactions with photoperiod sensitivity for maize latitude adaptation. Based on the analysis of PSIs in the study, a total of 48 QTLs and 252 significant SNPs were identified by linkage mapping and the GWAS approach, respectively. Five critical candidate genes, *MYB163*, *BIF1*, *BURP8*, *CADR3*, and *Zm00001d050238*, were significantly associated with photoperiod sensitivity, which could be deeply studied in further research. These results may provide much more abundant theoretical proof to reveal the genetic basis of photoperiod sensitivity, which would be beneficial for tropical germplasm improvement in current maize molecular design-based breeding.

## Data Availability

The original contributions presented in the study are included in the article/**Supplementary Material**, further inquiries can be directed to the corresponding author/s.

## Supplementary material

Supplementary Figure 1 The QTL mapping identified in this population.

Supplementary Figure 2 GO **(A)** and KEGG **(B)** analyses for the candidate genes identified by QTLs.

Supplementary Figure 3 GO enrichment analysis and pathway prediction for candidate genes identified by GWAS. The significant GO terms were selected by *p*-values at the 0.001 level.

Supplementary Figure 4 The pathway prediction of corporate DEGs identified by transcriptome data through the use of GO enrichment analysis.

Supplementary Figure 5 The pathway prediction of corporately downregulated DEGs identified by transcriptome data through the use of GO enrichment analysis.
