# Supplementary figures and images for: Integrated GWAS, linkage, and transcriptome analysis to identify genetic loci and candidate genes for photoperiod sensitivity in maize
Source: Front Plant Sci. 2024 Sep 16;15:1441288. doi: 10.3389/fpls.2024.1441288 (PMC11440433; doi:10.3389/fpls.2024.1441288)

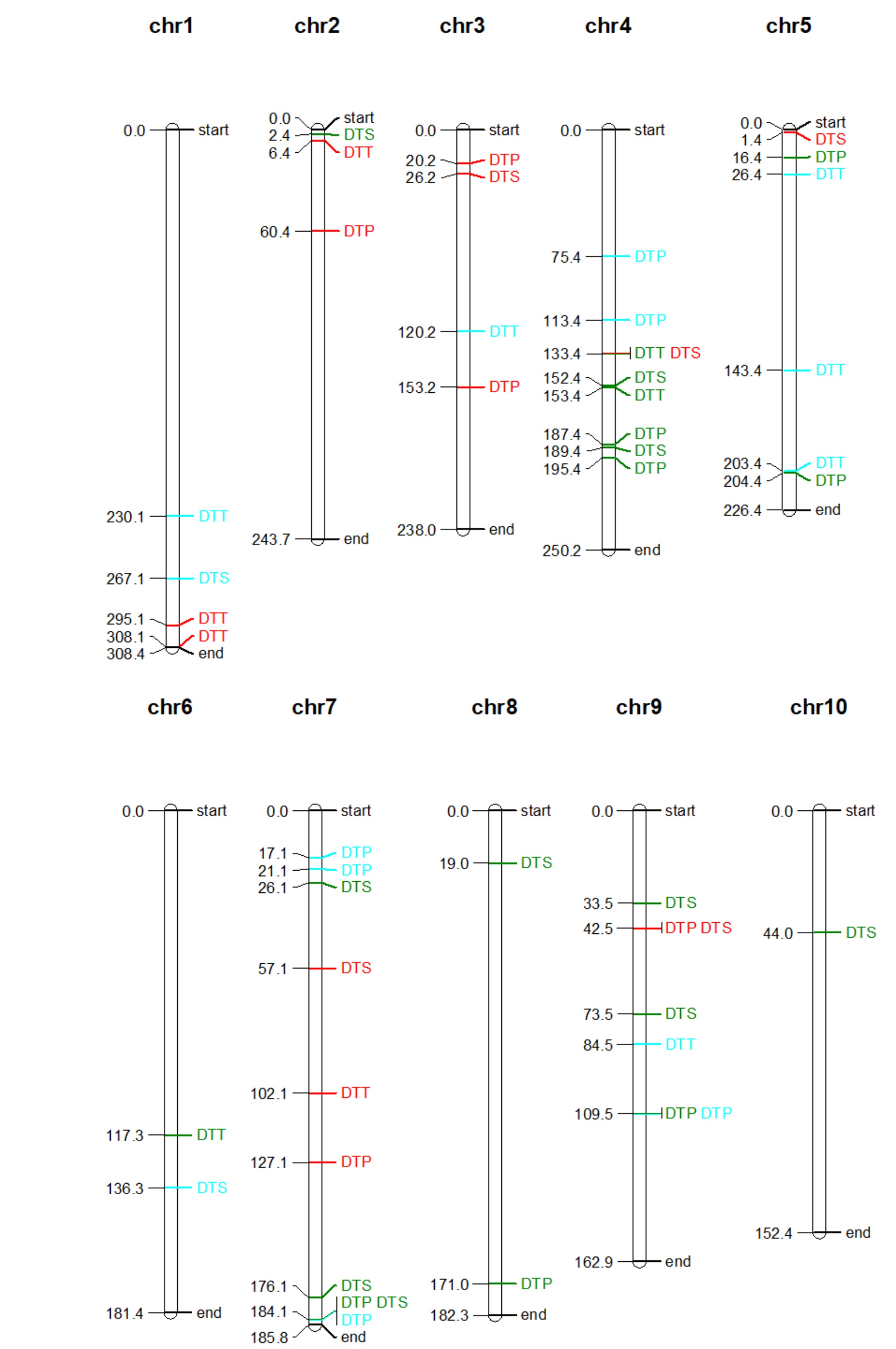

Supplement: Supplementary Figure 1 — The QTL mapping identified in this population. [file Image1.jpeg]

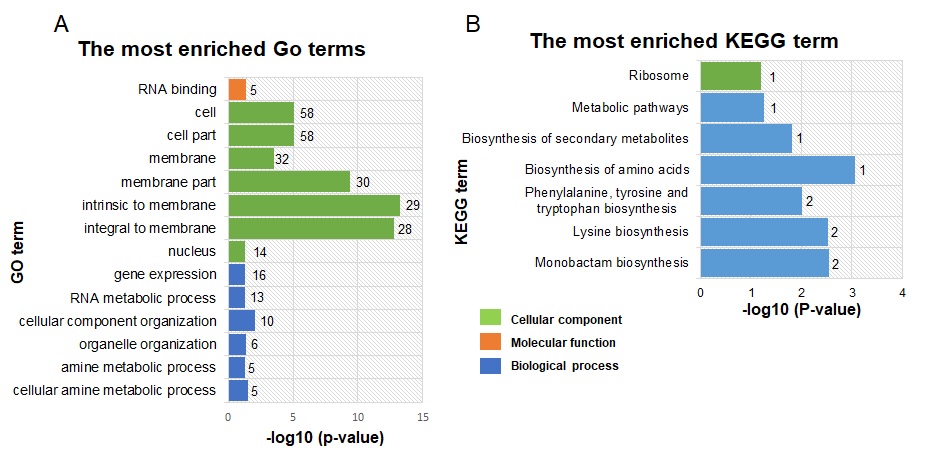

Supplement: Supplementary Figure 2 — GO (A) and KEGG (B) analyses for the candidate genes identified by QTLs. [file Image2.jpeg]

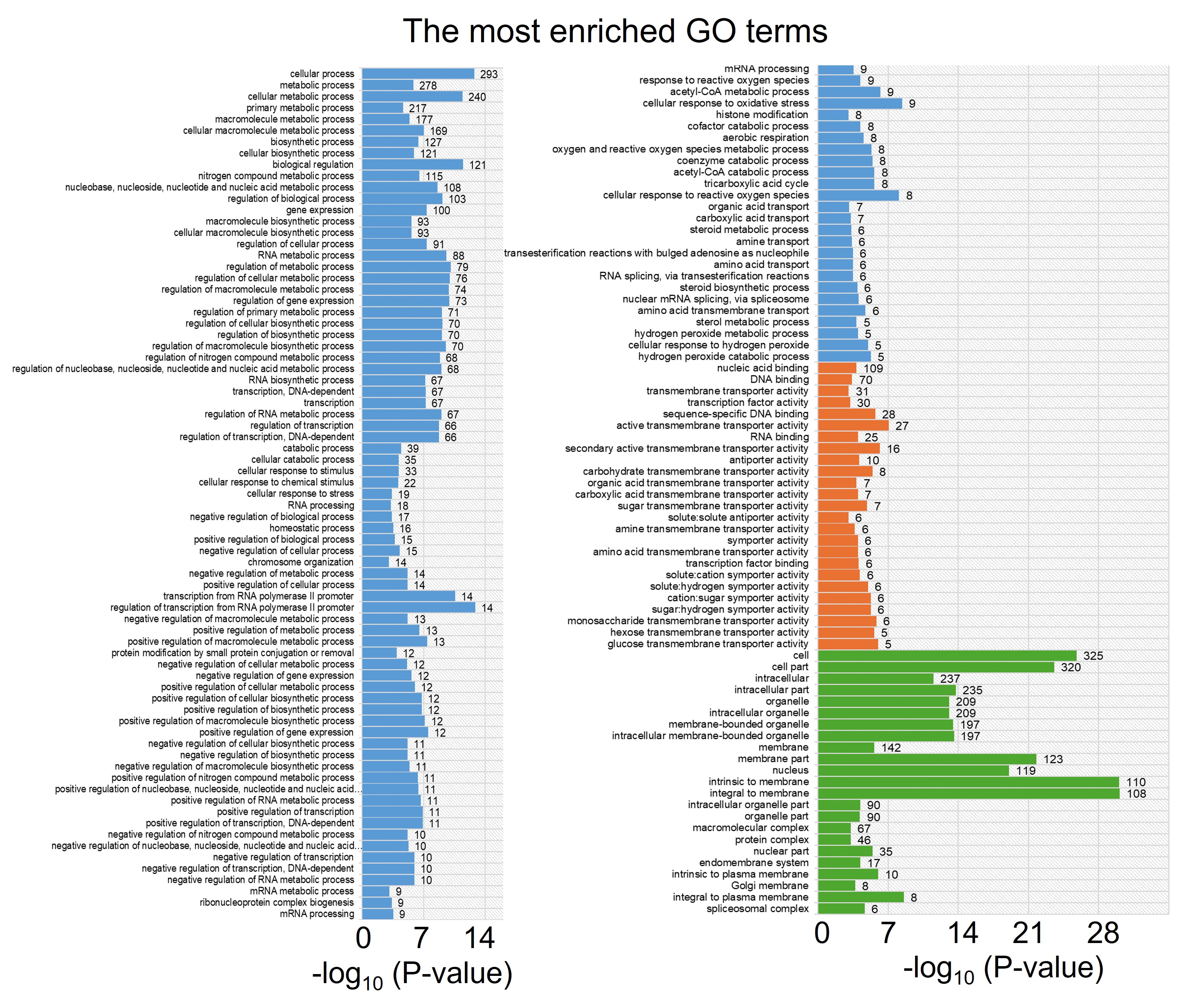

Supplement: Supplementary Figure 3 — GO enrichment analysis and pathway prediction for candidate genes identified by GWAS. The significant GO terms were selected by p-values at the 0.001 level. [file Image3.jpeg]

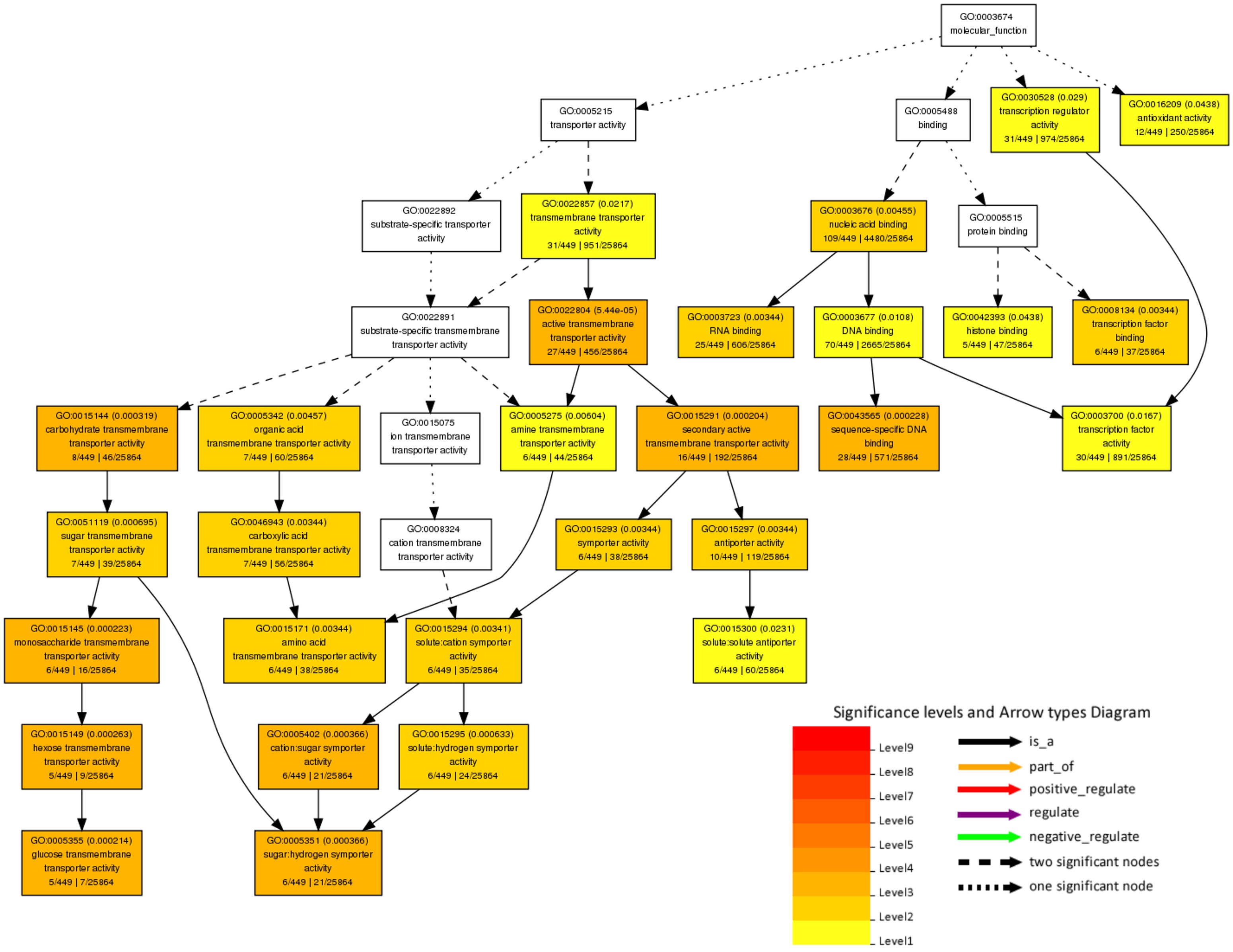

Supplement: Supplementary Figure 4 — The pathway prediction of corporate DEGs identified by transcriptome data through the use of GO enrichment analysis. [file Image4.jpeg]

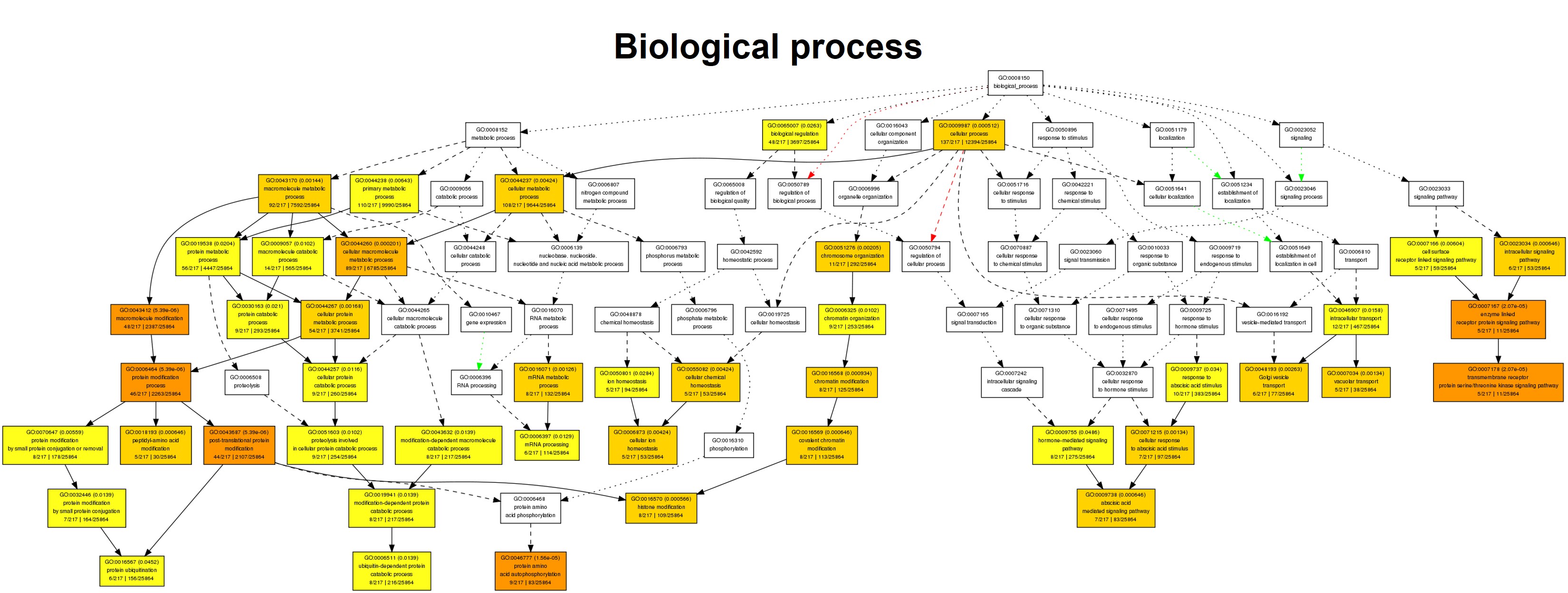

Supplement: Supplementary Figure 5 — The pathway prediction of corporately downregulated DEGs identified by transcriptome data through the use of GO enrichment analysis. [file Image5.jpeg]
